# Comparison of weekly docetaxel regimens in prostate cancer: a systematic review and frequentist network meta-analysis

**DOI:** 10.37349/etat.2026.1002360

**Published:** 2026-02-27

**Authors:** Shree Rath, Fatima Sajjad, Khawaja Abdul Rehman, Zaryab Bacha, Ahmad Omar Saleh, Umair Hayat, Umama Alam, Waseef Ullah, Fareeda Brohi, Osama Ahmad, Muzamil Khan, Amar Lal

**Affiliations:** IRCCS Istituto Romagnolo per lo Studio dei Tumori (IRST) “Dino Amadori”, Italy; ^1^Department of Medicine, All India Institute of Medical Sciences, Bhubaneswar 751019, India; ^2^Department of Medicine, Khyber Medical College, Peshawar 25000, Pakistan; ^3^Department of Medicine, CMH Lahore Medical College and Institute of Dentistry, Lahore 54000, Pakistan; ^4^Department of Medicine, The University of Jordan, Amman 11942, Jordan; ^5^Department of Medicine, Lady Reading Hospital, Peshawar 25000, Pakistan; ^6^Department of Medicine, Peoples University of Medical and Health Sciences for Women, Nawabshah 67450, Pakistan; ^7^Department of Medicine, The George Washington University School of Medicine and Health Sciences, Washington, DC 20052, USA; ^8^Department of Medicine, Penn State Health Milton S Hershey Medical Center, Hershey, PA 17033, USA

**Keywords:** docetaxel, prostate cancer, network meta-analysis, dosing

## Abstract

**Background:**

Docetaxel is a cornerstone chemotherapy for metastatic hormone-sensitive and castration-resistant prostate cancer. Although the standard triweekly regimen is widely used, weekly and biweekly schedules are often employed to improve tolerability, particularly in elderly or frail patients. The comparative efficacy and safety of these dosing strategies remain unclear. This study aimed to systematically compare weekly, biweekly, and triweekly docetaxel regimens using a network meta-analysis.

**Methods:**

MEDLINE, EMBASE, and the Cochrane Central Register of Controlled Trials were searched from inception to February 2025. Randomized controlled trials and observational retrospective studies comparing weekly, biweekly, and triweekly docetaxel regimens were included. Outcomes assessed were prostate-specific antigen (PSA) response rate, time to treatment failure or progression, and adverse events. A frequentist random-effects network meta-analysis was conducted using R software.

**Results:**

Eleven studies involving 1,238 patients were included. PSA response rates did not differ significantly among regimens; triweekly docetaxel showed a numerically lower response compared with weekly dosing (RR = 0.79, 95% CI 0.52–1.22; *I^2^* = 41.1%). Time to treatment failure was significantly longer with triweekly dosing compared with weekly dosing (mean difference = 10.91 months, 95% CI 6.94–14.87; *I^2^* = 96.8%). Biweekly and triweekly regimens were associated with significantly higher hepatotoxicity compared with weekly dosing (RR = 3.71 and RR = 3.21, respectively; *I^2^* = 0%). Vomiting was more frequent with triweekly docetaxel (RR = 2.47, 95% CI 1.31–4.63). No significant differences were observed for overall adverse events, hematologic toxicity, neuropathy, fatigue, febrile neutropenia, nausea, anorexia, or diarrhea.

**Discussion:**

Docetaxel dosing schedules show comparable PSA response rates. Triweekly dosing prolongs time to treatment failure but is associated with greater toxicity, whereas weekly dosing offers better tolerability. Treatment decisions should balance efficacy and safety based on individual patient characteristics.

## Introduction

Prostate cancer ranks as the second most prevalent cancer among men globally and is the fifth leading cause of death due to cancer, with around 1.4 million new diagnoses each year [1]. Systemic treatments, such as androgen deprivation therapy (ADT) and chemotherapy, play a vital role in the management of metastatic hormone-sensitive prostate cancer (mHSPC) as well as metastatic castration-resistant prostate cancer (mCRPC) [2].

Docetaxel, a microtubule inhibitor derived from the taxane class, has emerged as a fundamental therapy [3]. In mCRPC, the combination of docetaxel and prednisone has demonstrated a survival advantage of 20–24% compared to treatment regimens based on mitoxantrone [4]. In the context of mHSPC, pivotal studies like CHAARTED and STAMPEDE have shown that the integration of docetaxel with ADT markedly enhances overall survival rates, particularly among patients presenting with high-volume disease [5].

Although the standard docetaxel regimen involves triweekly administration (75 mg/m^2^ every 3 weeks), alternative weekly (30 mg/m^2^) and biweekly (50 mg/m^2^) schedules have been explored to enhance tolerability and reduce toxicity, especially in frail or elderly patients [6]. Weekly and biweekly regimens have shown potential to lower adverse events, such as febrile neutropenia, while maintaining efficacy comparable to the triweekly schedule in mCRPC [7]. A recent retrospective study examining weekly, biweekly, and triweekly docetaxel treatment regimens in patients with mCRPC revealed no significant differences in prostate-specific antigen (PSA) response rates among the three dosing frequencies. Nevertheless, both biweekly and triweekly regimens demonstrated improved overall survival compared to the weekly regimen. Additionally, the time to disease progression was marginally extended with the less frequent dosing schedules. Notably, the rates of overall and high-grade toxicities were comparable across all treatment groups [8]. A randomized trial in mCRPC reported that a biweekly 50 mg/m^2^ regimen resulted in longer time to treatment failure and fewer severe toxicities compared to the triweekly 75 mg/m^2^ regimen [9]. These findings suggest that alternative dosing schedules may offer a better balance of efficacy and safety, particularly for patients with comorbidities.

However, the optimal weekly docetaxel regimen for prostate cancer remains unclear due to limited head-to-head comparisons and heterogeneity in study designs, patient populations, and outcome measures [10]. Despite extensive investigation of docetaxel-based therapy in prostate cancer, several clinically important gaps remain unresolved. Most available trials were not designed to inform individualized dosing strategies, and treatment selection is often guided by convention rather than patient-specific factors such as age, comorbidities, performance status, or frailty. Elderly patients and those with significant comorbid conditions, who represent a substantial proportion of the real-world prostate cancer population, are frequently underrepresented in randomized trials, limiting the applicability of standard triweekly dosing in routine practice [9, 10]. Additionally, direct head-to-head comparisons among weekly, biweekly, and triweekly regimens are scarce, and existing meta-analyses have primarily relied on pairwise comparisons rather than integrated evidence synthesis. In this context, network meta-analysis (NMA) offers an effective method for integrating both direct and indirect evidence, facilitating a thorough comparison of various treatment regimens at once and aiding in the selection of regimens tailored to individual patient characteristics [11].

To our knowledge, no previous NMA has systematically evaluated and compared the efficacy and safety of weekly, biweekly, and triweekly docetaxel regimens in prostate cancer [12]. This study aims to address this gap by conducting a systematic review and NMA of randomized controlled trials (RCTs), retrospective studies, and case-control studies. We will evaluate clinical outcomes such as PSA response rates and adverse events associated with various dosing regimens. The results of our study are intended to inform personalized treatment strategies and improve therapeutic results for men diagnosed with prostate cancer.

## Materials and methods

This systematic review and meta-analysis were carried out in compliance with the guidelines given in the Cochrane Handbook for Systematic Reviews of Interventions [13] and reported according to the Preferred Reporting Items for Systematic Reviews and Meta-Analyses (PRISMA) guidelines [14]. The study protocol has been registered with the International Prospective Register of Systematic Reviews (PROSPERO) CRD420251076649.

### Data sources and searches

A systematic literature search was undertaken on three databases: MEDLINE, EMBASE, and Cochrane Central Register of Controlled Trials using MESH terms and keywords related to [Prostate Cancer] and [Docetaxel] from inception till February 2025. During screening, we did not use any filter based on author, title, study type, or year of publication. In order to enhance the diversity of our study, a supplementary search was conducted by reviewing the references of all included articles. The detailed search strategy is provided in Table S1.

### Study selection and eligibility criteria

All studies were imported to Rayyan, and with the help of a filter, all duplicates generated by our literature search were removed. Screening of titles and abstracts was carried out independently by two authors (OA and FS) to exclude all irrelevant studies. Full-text screening was then carried out on the remaining studies in compliance with our eligibility criteria. All disputes regarding the selection of studies were resolved by a third author (SR).

All observational studies and RCTs that compare weekly, biweekly, and triweekly docetaxel regimens in patients with histologically or cytologically confirmed prostate cancer were included in this meta-analysis. Those patients were excluded who had no complete information available or who did not match the inclusion criteria. Study designs other than cohorts, case-control studies, and RCTs were excluded. Studies conducted on animals, case reports, reviews, and systematic reviews and meta-analyses were also excluded. No language or date restrictions were applied.

### Data extraction and outcomes

All relevant data were extracted into an Excel sheet comprised of three domains. The first domain consists of the author’s name, study design, inclusion and exclusion criteria, duration of follow-up, intervention name, dosing, and route of administration. The second domain comprised baseline characteristics, and the third one reported outcome data. Data extraction was independently carried out by two authors, and conflicts were resolved by a third author. The primary outcomes of this study included PSA response rate and time for treatment failure or progression. The secondary outcomes comprised hepatotoxicity, vomiting, any adverse event, thrombocytopenia, anemia, arthralgia, neuropathy, neutropenia, fatigue, febrile neutropenia, nausea, anorexia, and diarrhea.

### Risk of bias assessment

The methodological quality of included studies was evaluated using design-specific appraisal tools. RCTs were assessed with the revised Cochrane risk of bias tool for randomized trials (RoB 2) [15], which examines potential bias across five domains: the randomization process, deviations from intended interventions, completeness of outcome data, outcome measurement, and selective reporting. Based on these domains, each trial was assigned an overall judgment of low risk of bias, some concerns, or high risk of bias.

Observational studies were appraised using the Newcastle-Ottawa Scale (NOS) for observational research [16], which evaluates study quality across three domains: selection of participants, comparability of study groups, and assessment of outcomes. To facilitate interpretation, NOS scores were mapped to Agency for Healthcare Research and Quality (AHRQ) categories of good, fair, or poor quality. The maximum achievable scores were four stars for selection, two for comparability, and three for outcome assessment. Studies receiving 7–9 stars were classified as low risk of bias, those with 5–6 stars as having some concerns, and those scoring fewer than 5 stars as high risk of bias. All assessments were conducted independently by two reviewers (AK and UJ), and any discrepancies were resolved through consensus or by involving a third reviewer (AA), ensuring a rigorous and transparent quality appraisal process.

### Statistical analysis

The NMA was conducted using the netmeta package of the R statistical tool (R Development Core Team), version 3.2.3. A frequentist random-effects model was used in the NMA to compare the efficacy of weekly (referred to as 1), biweekly (referred to as 2), and triweekly (referred to as 3) docetaxel therapy using the netmeta package of R. Dichotomous outcomes were analyzed using risk ratios (RRs) and 95% confidence intervals (CIs) through a frequentist NMA. Both direct and indirect effect sizes were calculated, and global and local inconsistencies were assessed (with statistical significance for inconsistencies set at < 0.05). Forest plots were created using the common comparator of control to demonstrate network estimates (RRs, CIs, and *p*-values). Data was demonstrated in the form of league tables containing network estimates for all possible comparisons. Each intervention’s probability of being the most effective was ranked based on probability-scores (P-scores) values. Heterogeneity was assessed using the DerSimonian-Laird estimator for *tau^2^*. All analyses were performed using R version 4.4.1, and a *p*-value of less than 0.05 was considered statistically significant.

## Results

### Searched results

**Figure 1:**
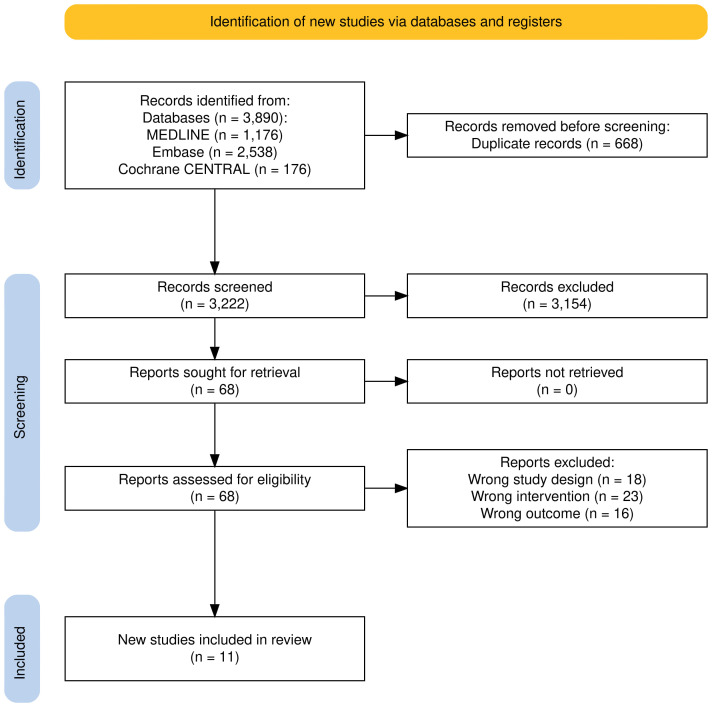
**PRISMA flowchart of screening and study inclusion.** PRISMA: Preferred Reporting Items for Systematic Reviews and Meta-Analyses. Adapted from [14]. © Author(s) 2021. CC BY 4.0.

Initially, an electronic search yielded a total of 3,890 records, with 668 duplicates removed. In the subsequent screening phase, 3,154 records were excluded based on title and abstract evaluations. After reviewing 68 records, 18 were excluded due to the wrong study design, 23 records were excluded due to the wrong intervention, and 16 records were excluded due to the wrong outcomes. Overall, 4 RCTs [9, 17–19] and 7 retrospective studies were included [7, 8, 20–24]. The selection process is detailed in the PRISMA flowchart (Figure 1).

### Study characteristics

Of the 11 studies included, 4 were RCTs, and 7 were retrospective studies. The mean age was 69.21, ranging from 66.4 to 78 years in the biweekly docetaxel. In the triweekly docetaxel, the mean age was 68.10 years, ranging from 64.75 to 70 years. In the weekly docetaxel, the mean age was 69.59 years, ranging from 65.5 to 72.4 years. The mean PSA at baseline was 233.42 ng/mL (ranging from 31.1 to 646.775) in the biweekly docetaxel, 347.50 ng/mL (ranging from 34.7 to 888.275) in the triweekly docetaxel, and 169.1625 ng/mL (ranging from 90 to 237.1) in the weekly docetaxel.

According to the site of metastasis. The bone metastasis was reported in eight studies, with a mean percentage of 82.213% (ranging from 24 to 100%) in the triweekly docetaxel, 72.93% (ranging from 24 to 96.15%) in the biweekly docetaxel, and 87.35% (ranging from 76 to 97.5%) in the weekly docetaxel. The lung metastasis was reported in five studies, with a mean percentage of 37.6% (ranging from 9.8 to 79%) in biweekly docetaxel, 39.45% (ranging from 2.4 to 89%) in triweekly docetaxel, and 32.37% (ranging from 2.7 to 76%) in weekly docetaxel. The lymph node metastasis was reported in five studies, with a mean percentage of 49.3% (ranging from 24 to 79%) in triweekly docetaxel, 62.8% (ranging from 24 to 85.4%) in biweekly docetaxel, and 41.7% (ranging from 16.2 to 78.9%) in weekly docetaxel. The summary and baseline characteristics of the included studies are presented in Table 1.

**Table 1:** Baseline characteristics across included studies.

**Study**	**Design**	**Intervention**	**Control**	**Age (Intervention)**	**Age (Control)**	**Baseline PSA (Intervention)**	**Baseline PSA (Control)**	**ECOG-0 *n* (%)**	**ECOG-1 *n* (%)**	**ECOG-2 *n* (%)**	**ECOG-3 *n* (%)**
Shimura, 2020	Retrospective cohort	2-weekly docetaxel (25–35 mg/m^2^)	3-weekly docetaxel (60–75 mg/m^2^)	67.0 (8.08)	70.8 (8.33)	301.0 (526.9)	280.1 (499.8)	0 (0)/0 (0)	20 (76.9)/17 (65.4)	4 (15.4)/8 (30.8)	2 (7.7)/1 (3.8)
Hervonen, 2012	RCT	3-weekly docetaxel (75 mg/m^2^)	2-weekly docetaxel (50 mg/m^2^)	70.0 (8.82)	68.0 (7.98)	109.0 (200.3)	98.0 (310.6)	NR	25 (65)/20 (51)	NR	NR
Martinez-Recio, 2022	Retrospective study	3-weekly docetaxel (75 mg/m^2^)	2-weekly docetaxel (50 mg/m^2^)	69.0 (7.53)	78.0 (5.06)	888.3 (631.5)	646.8 (564.2)	NR	NR	NR	NR
Berthold, 2008	Retrospective study	3-weekly docetaxel + mitoxantrone	Weekly docetaxel + mitoxantrone	64.8 (0.61)	65.5 (0.42)	620.2 (459.7)	224.8 (132.3)	NR	NR	NR	NR
Kim, 2017	Retrospective study	3-weekly docetaxel	Biweekly docetaxel	69.1 (7.86)	66.5 (6.01)	34.7 (NR)	31.1 (NR)	NR	NR	NR	NR
Lehtonen, 2022	Phase III trial	3-weekly docetaxel	Biweekly docetaxel	66.8 (7.24)	67.5 (7.76)	116 (Median)	109 (Median)	NR	NR	NR	NR
Petrioli, 2011	Phase II RCT	Weekly docetaxel	3-weekly docetaxel	69.3 (7.29)	69.8 (6.42)	90.0 (39.5)	99.0 (57.5)	8 (21.6)/11 (31.1)	29 (78.4)/24 (68.6)	NR	NR
Yuk, 2024	Retrospective cohort	Weekly docetaxel	Biweekly/Triweekly docetaxel	72.4 (7.9)	70.2–68.2 (8.8–8.9)	237.1 (87.4)	714.3–778.7 (207.8–144)	11 (28.9)/17 (52.0)	8 (21.1)/16 (39.0)	4 (10.5)/0 (0)	15 (39.5)/8 (19.5)
Kellokumpu-Lehtinen, 2013	Phase III RCT	2-weekly docetaxel	3-weekly docetaxel	67.8 (7.27)	68.8 (7.80)	119.3 (42.15)	112.1 (36.03)	NR	NR	NR	NR
Samar, 2020	Retrospective study	2-weekly docetaxel	3-weekly docetaxel	67.3 (8.9)	67.3 (8.9)	236.2 (126.3)	294.1 (520.9)	5 (55.5)/3 (20.0)	3 (33.3)/8 (53.3)	1 (11.1)/2 (13.3)	0 (0)/2 (13.3)
Sayin, 2022	Retrospective study	3-weekly docetaxel	Weekly docetaxel	NR	NR	NR	NR	35 (89.7)/8 (20.0)	NR	NR	NR

RCT: randomized controlled trial; ECOG: Eastern Cooperative Oncology Group; PSA: prostate-specific antigen; NR: not reported; mg/m^2^: milligrams per square meter.

### Risk of bias and quality assessment of included studies

The quality of the four selected RCTs was assessed using RoB 2. The overall risk of bias was low in (2/4), with some concerns in (5/11), and with high risk in (3/11). Domain 1, the risk of bias arising from the randomization process, showed some concern in (2/4). Domain 4, the risk of bias in measuring the outcome, revealed a high risk in (1/4) (Figures S1 and S2). The quality of seven retrospective studies was assessed using the NOS. The overall quality rating of the included studies was 9 out of 9 stars in two studies, 7 out of 9 stars in four studies, and 6 out of 9 stars in one study. Table S2 represents the NOS of the included studies.

### Outcomes

#### Primary outcomes

##### PSA response rate

**Figure 2:**
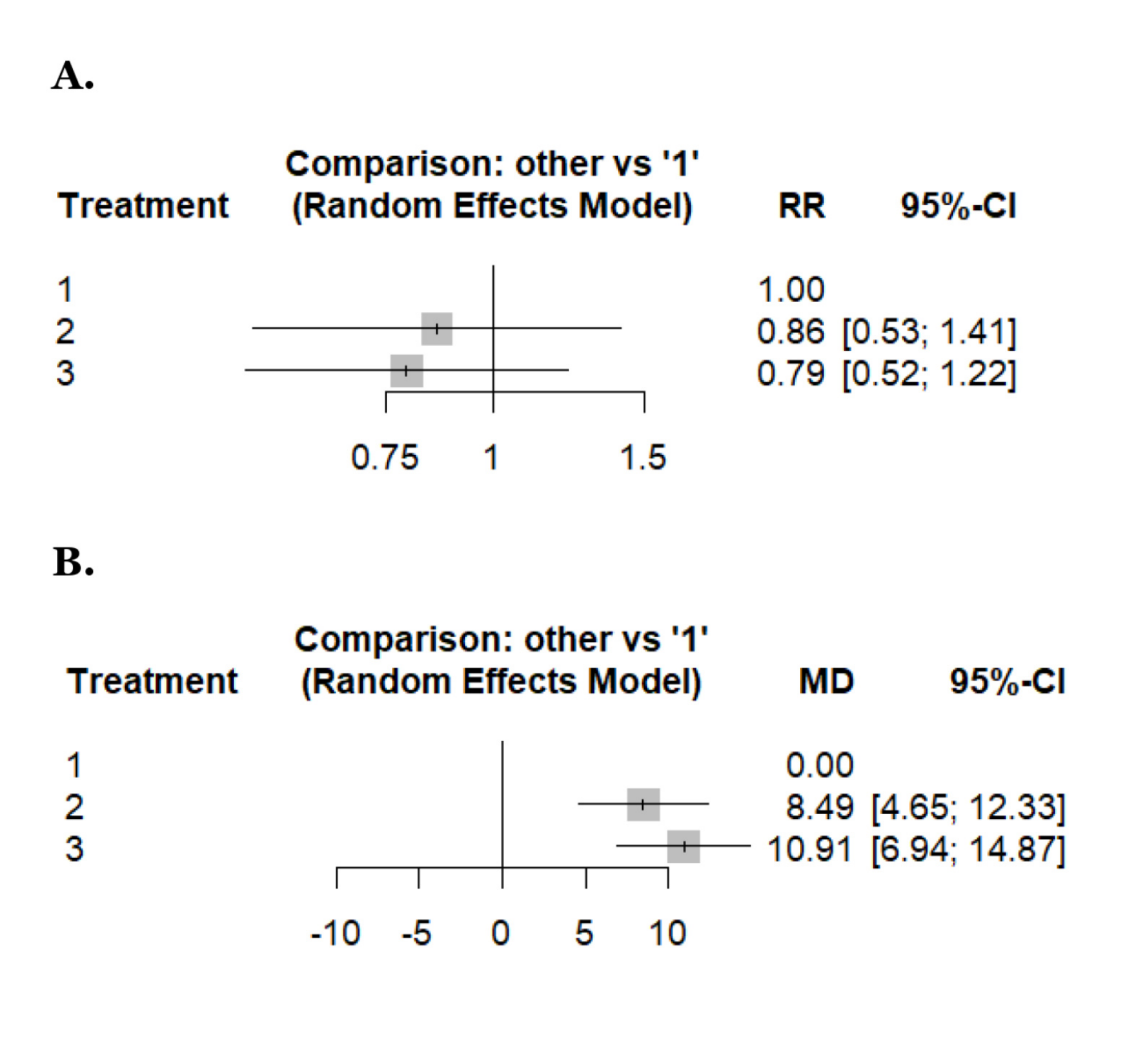
**Forest plots reporting results of the network meta-analysis concerning: (A) PSA response rate, (B) time to treatment failure/progression.** Treatment regimens: 1: weekly, 2: biweekly, 3: triweekly. PSA: prostate-specific antigen.

An NMA of the PSA response rate (5 studies, 757 patients, and three treatment regimens assessed) showed a lower PSA response rate on using triweekly (RR: 0.79; 95% CI: 0.52–1.22; *p* = 0.23) or biweekly docetaxel (RR: 0.86; 95% CI: 0.53–1.41; *p* = 0.48) as compared to weekly docetaxel, although the difference failed to reach statistical significance (Figure 2A). Weekly docetaxel therapy was ranked as the most effective intervention based on P-scores (0.79), followed by biweekly (0.48) and triweekly (0.23). The evidence structure was assessed via a netweight plot, which revealed that the estimates for most comparisons were predominantly supported by direct evidence (Figure S3). Specifically, the comparison between biweekly and triweekly was informed by 96% direct evidence, while the biweekly vs. weekly estimate relied on a higher proportion (58%) of indirect evidence (Figure S4). Despite this, the netheat plot, along with global and local assessments of inconsistency (*p* = 0.1475 and all local *p* > 0.05, respectively), confirmed the reliability of the network estimates and the validity of the transitivity assumption (Figure S5). Upon visual inspection of the comparison-adjusted funnel plot, no clear evidence of publication bias was observed, although the small number of studies (*n* < 10) remains a limitation (Figure S6).

##### Time for treatment failure or progression

With weekly docetaxel as the reference, both biweekly and triweekly docetaxel were associated with a significantly longer time to treatment failure or progression. The pooled network estimates showed an increase in time to failure/progression for biweekly docetaxel (MD: 8.49; 95% CI: 4.65–12.33) and for triweekly docetaxel (MD: 10.91; 95% CI: 6.94–14.87) compared with weekly dosing (Figure 2B). According to P-scores, weekly docetaxel ranked as the most favorable schedule for minimizing time to treatment failure or progression (P-score: 0.99), followed by biweekly docetaxel (P-score: 0.51), while triweekly docetaxel ranked lowest (P-score: 0.00) (Figure S7). Assessment of the netweight plot demonstrated that the majority of network estimates were predominantly supported by direct evidence. The comparison between biweekly and triweekly docetaxel was informed entirely by direct evidence (100%), whereas the weekly vs. biweekly and weekly vs triweekly comparisons incorporated a larger proportion of indirect evidence, reflecting the limited number of direct head-to-head trials involving weekly dosing (Figure S8). No specific comparison was identified as contributing meaningfully to inconsistency, indicating that the pooled network estimates are robust. Visual inspection of the comparison-adjusted funnel plot did not show marked asymmetry, suggesting no clear evidence of publication bias, although this finding should be interpreted cautiously given the small number of included studies (Figure S9).

#### Secondary outcomes

**Figure 3:**
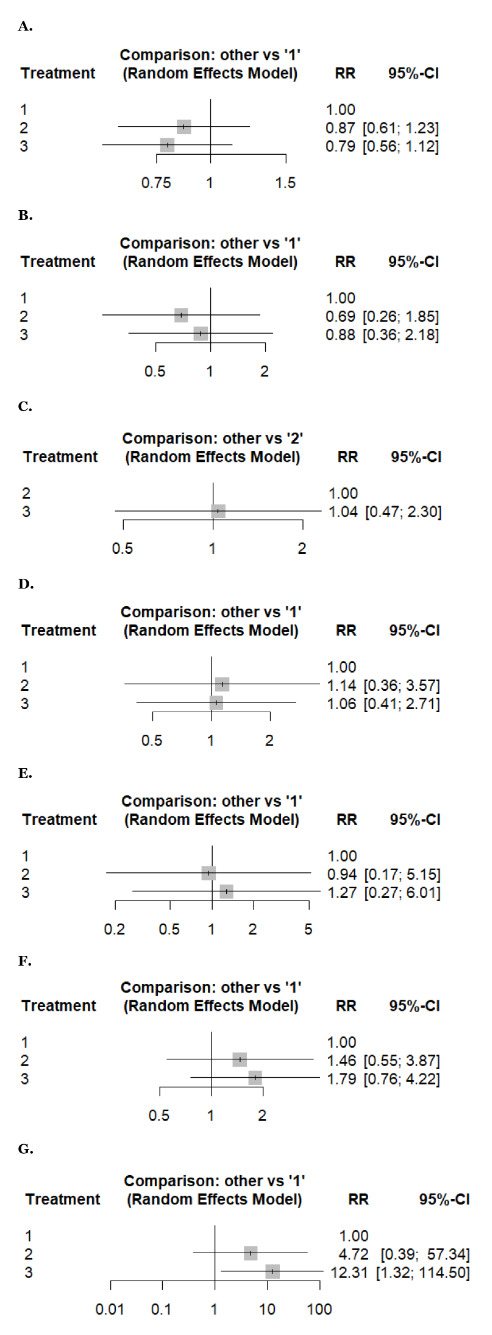
**Forest plots reporting results of the network meta-analysis of: (A) any adverse events, (B) anemia, (C) anorexia, (D) arthralgia, (E) diarrhea, (F) fatigue, (G) febrile neutropenia.** Treatment regimens: 1: weekly, 2: biweekly, 3: triweekly.

**Figure 4:**
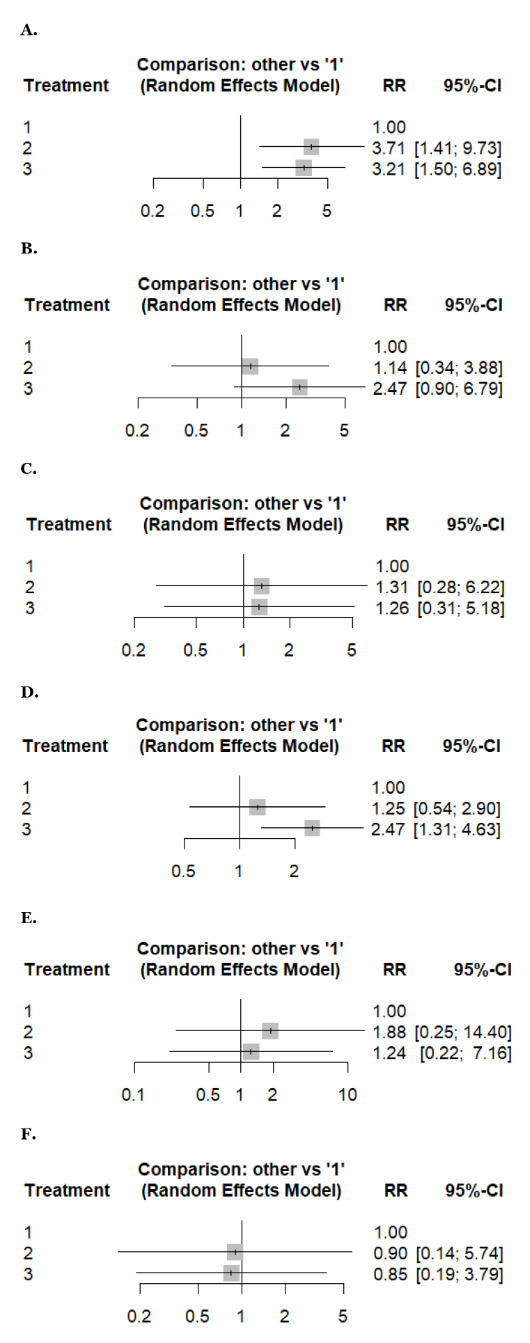
**Forest plots reporting results of the network meta-analysis of: (A) hepatotoxicity, (B) nausea, (C) neuropathy, (D) vomiting, (E) neutropenia, (F) thrombocytopenia.** Treatment regimens: 1: weekly, 2: biweekly, 3: triweekly.

Figures 3 and 4 present forest plots reporting the results of the NMA.

##### Hepatotoxicity and vomiting

An NMA of hepatotoxicity (2 studies, 241 patients, and three treatment regimens assessed) and vomiting (3 studies, 618 patients, and three treatment regimens assessed). Compared with weekly docetaxel, biweekly docetaxel showed a statistically significantly higher risk of hepatotoxicity (RR: 3.71, 95% CI 1.41–9.73), followed by triweekly docetaxel (RR: 3.21, 95% CI 1.50–6.89) with low heterogeneity (*I^2^* = 0%) (Figure 4A). However, triweekly docetaxel has a higher risk of vomiting (RR: 2.47, 95% CI 1.31–4.63) with statistical significance compared with weekly docetaxel with low heterogeneity (*I^2^* = 0%) (Figure 4D).

##### Any adverse events and thrombocytopenia

An NMA of any adverse events (2 studies, 359 patients, and three treatment regimens assessed) and thrombocytopenia (4 studies, 341 patients, and three treatment regimens assessed). Compared to weekly docetaxel, biweekly regimens carried a higher risk (RR: 0.87, 95% CI 0.61–1.23) and (RR: 0.90, 95% CI 0.14–5.74), respectively, followed by triweekly (RR: 0.79, 95% CI 0.56–1.12) and (RR: 0.85, 95% CI 0.19–3.79), respectively. However, the difference was insignificant, with low and moderate heterogeneity (*I^2^* = 0% and 44.18%) (Figure 3A and Figure 4F).

##### Anemia

An NMA of anemia (5 studies, 863 patients, and three treatment regimens assessed). Weekly docetaxel showed a higher risk of anemia (RR: 1) than triweekly (RR: 0.88, 95% CI 0.36–2.18), and then biweekly (RR: 0.69, 95% CI 0.26–1.85) without statistical significance, with high heterogeneity (*I^2^* = 91.1%) (Figure 3B).

##### Arthralgia, neuropathy, and neutropenia

An NMA of arthralgia (3 studies, 254 patients, and three treatment regimens assessed), neuropathy (5 studies, 637 patients, and three treatment regimens assessed), and neutropenia (5 studies, 863 patients, and three treatment regimens assessed). Compared to weekly docetaxel, biweekly docetaxel showed a higher risk of arthralgia, neuropathy, and neutropenia (RR: 1.14, 95% CI 0.36–3.57), (RR: 1.31, 95% CI 0.28–6.22), and (RR: 1.88, 95% CI 0.25–14.40), respectively, followed by triweekly docetaxel (RR: 1.06, 95% CI 0.41–2.71), (RR: 1.26, 95% CI 0.31–5.18), and (RR: 1.24, 95% CI 0.22–7.16), respectively. However, the difference was insignificant with low, moderate, and high heterogeneity, respectively (*I^2^* = 0%, 34.1%, and 79.4%) (Figure 3D, Figure 4C, and Figure 4E).

##### Fatigue, febrile neutropenia, and nausea

An NMA of fatigue (7 studies, 1,238 patients, and three treatment regimens assessed), febrile neutropenia (4 studies, 376 patients, and three treatment regimens assessed), and nausea (4 studies, 666 patients, and three treatment regimens assessed). Compared to weekly docetaxel, triweekly docetaxel showed a higher risk of fatigue, febrile neutropenia, and nausea (RR: 1.79, 95% CI 0.76–4.22), (RR: 12.3, 95% CI 1.32–114.50), and (RR: 2.47, 95% CI 0.90–6.79), respectively, followed by biweekly docetaxel (RR: 1.46, 95% CI 0.55–3.87), (RR: 4.72, 95% CI 0.39–57.34), and (RR: 1.14, 95% CI 0.34–3.88), respectively. However, the difference was insignificant with high, low, and moderate heterogeneity (*I^2^* = 93%, 22.3%, and 38.6%) (Figure 3F, Figure 3G, and Figure 4B).

##### Anorexia and diarrhea

An NMA of anorexia (2 studies, 182 patients, and two treatment regimens assessed) and diarrhea (5 studies, 824 patients, and three treatment regimens assessed). Regarding anorexia, triweekly docetaxel showed a higher risk (RR: 1.04, 95% CI 0.47–2.30) as compared to biweekly docetaxel without statistical significance with low heterogeneity (*I^2^* = 0%) (Figure 3C). While triweekly docetaxel has a higher risk of diarrhea (RR: 1.27, 95% CI 0.27–6.01), followed by weekly docetaxel (RR: 1), and then biweekly docetaxel (RR: 0.94, 95% CI 0.17–5.15), without statistical significance with high heterogeneity (*I^2^* = 70.9%) (Figure 3E).

League tables of pairwise and NMA are presented in Table 2, and local inconsistency across outcomes is detailed in Table S3.

**Table 2:** League tables presenting the pairwise meta-analysis (upper-right half of the table) and the network meta-analysis (lower-left half of the table) differences among docetaxel doses in prostate cancer for all outcomes.

**Outcome**	**Treatment comparison**		
PSA response rate	Biweekly	1.13 (0.82; 1.57)	0.79 (0.37; 1.70)
	1.08 (0.79; 1.49)	Triweekly	0.76 (0.48; 1.19)
	0.86 (0.53; 1.41)	0.79 (0.52; 1.22)	Weekly
Time for treatment failure or progression*	Biweekly	**–2.42 (–4.65; –0.19)**	3.20 (–1.15; 7.55)
	**–2.42 (–4.65; –0.19)**	Triweekly	**18.80 (13.79; 23.81)**
	**8.49 (4.65; 12.33)**	**10.91 (6.94; 14.87)**	Weekly
Hepatotoxicity	Biweekly	1.21 (0.58; 2.54)	2.78 (0.81; 9.51)
	1.15 (0.56; 2.38)	Triweekly	**3.21 (1.50; 6.89)**
	**3.71 (1.41; 9.73)**	**3.21 (1.50; 6.89)**	Weekly
Vomiting	Biweekly	**0.51 (0.29; 0.89)**	NA
	**0.51 (0.29; 0.89)**	Triweekly	**2.47 (1.31; 4.63)**
	1.25 (0.54; 2.90)	**2.47 (1.31; 4.63)**	Weekly
Any adverse events	Biweekly	1.09 (0.99; 1.21)	0.88 (0.58; 1.35)
	1.09 (0.99; 1.21)	Triweekly	0.78 (0.54; 1.14)
	0.87 (0.61; 1.23)	0.79 (0.56; 1.12)	Weekly
Thrombocytopenia	Biweekly	1.07 (0.36; 3.15)	NA
	1.07 (0.36; 3.15)	Triweekly	0.85 (0.19; 3.79)
	0.90 (0.14; 5.74)	0.85 (0.19; 3.79)	Weekly
Anemia	Biweekly	0.78 (0.53; 1.16)	NA
	0.78 (0.53; 1.16)	Triweekly	0.88 (0.36; 2.18)
	0.69 (0.26; 1.85)	0.88 (0.36; 2.18)	Weekly
Arthralgia	Biweekly	1.07 (0.56; 2.07)	NA
	1.07 (0.56; 2.07)	Triweekly	1.06 (0.41; 2.71)
	1.14 (0.36; 3.57)	1.06 (0.41; 2.71)	Weekly
Neuropathy	Biweekly	1.07 (0.51; 2.27)	0.31 (0.01; 8.34)
	1.04 (0.49; 2.19)	Triweekly	1.40 (0.33; 5.97)
	1.31 (0.28; 6.22)	1.26 (0.31; 5.18)	Weekly
Neutropenia	Biweekly	1.52 (0.54; 4.26)	NA
	1.52 (0.54; 4.26)	Triweekly	1.24 (0.22; 7.16)
	1.88 (0.25; 14.40)	1.24 (0.22; 7.16)	Weekly
Fatigue	Biweekly	0.85 (0.47; 1.53)	1.30 (0.26; 6.52)
	0.82 (0.46; 1.45)	Triweekly	1.66 (0.68; 4.05)
	1.46 (0.55; 3.87)	1.79 (0.76; 4.22)	Weekly
Febrile neutropenia	Biweekly	0.38 (0.12; 1.18)	NA
	0.38 (0.12; 1.18)	Triweekly	**12.31 (1.32; 114.50)**
	4.72 (0.39; 57.34)	**12.31 (1.32; 114.50)**	Weekly
Nausea	Biweekly	0.46 (0.24; 0.92)	NA
	0.46 (0.24; 0.92)	Triweekly	2.47 (0.90; 6.79)
	1.14 (0.34; 3.88)	2.47 (0.90; 6.79)	Weekly
Diarrhea	Biweekly	0.74 (0.37; 1.48)	NA
	0.74 (0.37; 1.48)	Triweekly	1.27 (0.27; 6.01)
	0.94 (0.17; 5.15)	1.27 (0.27; 6.01)	Weekly
Anorexia**	NA		

*: Mean difference (MD); all other outcomes report risk ratio (RR). **: No network estimate due to a single comparison of triweekly vs. biweekly therapy. NA represents that there is no direct evidence available for that specific comparison. Note: effect estimates (RR or MD) with 95% confidence intervals from the NMA are located at the intersection of each row- and column-defining intervention. In the lower-left half of the table, an RR < 1 (or MD < 0) favors the row-defining treatment, while in the upper-right half, an OR < 1 (or MD < 0) favors the column-defining treatment, with statistically significant results presented in bold. NMA: network meta-analysis.

## Discussion

This NMA evaluated and compared the efficacy and safety profiles of weekly, biweekly, and triweekly docetaxel regimens in patients with prostate cancer, using data from 11 RCTs and retrospective studies. Weekly docetaxel demonstrated the highest PSA response rate, while the triweekly regimen was associated with a significantly longer time to treatment failure. In terms of safety, biweekly and triweekly regimens showed a significantly higher incidence of hepatotoxicity and vomiting compared to weekly dosing. Weekly docetaxel had a higher incidence of anemia and thrombocytopenia, though these differences were not statistically significant. Triweekly dosing showed the highest rates of febrile neutropenia, fatigue, and nausea, whereas biweekly regimens were more frequently associated with arthralgia, neuropathy, and neutropenia. Most adverse events did not reach statistical significance and were characterized by varying degrees of heterogeneity. These findings highlight distinct risk-benefit profiles among the three regimens, providing valuable insights for tailoring docetaxel-based therapy in clinical practice.

Our NMA indicated that weekly docetaxel administration achieved the highest PSA response rate, while triweekly dosing exhibited the lowest, though these differences were not statistically significant. This finding is consistent with previous meta-analyses. For instance, a systematic review and meta-analysis by Neto et al. [25] evaluated 12 randomized clinical trials involving 2,244 patients and found no significant difference in PSA response rates between weekly and triweekly docetaxel regimens. Similarly, a meta-analysis by Francini et al. [26] analyzed 22 trials encompassing 7,677 patients and reported a moderate correlation between PSA response and overall survival, suggesting that variations in dosing schedules did not markedly impact PSA response rates. These studies support the notion that while weekly dosing may offer a marginally higher PSA response, the differences among the various schedules are not statistically significant, emphasizing the importance of considering patient-specific factors such as tolerability and comorbidities when selecting a docetaxel regimen.

Our analysis demonstrated that the triweekly docetaxel regimen was associated with a significantly longer time to treatment failure compared to the weekly regimen, with high heterogeneity. This finding is supported by a meta-analysis by Neto et al. [25], which evaluated 12 randomized trials and found that triweekly regimens were more effective in delaying disease progression than weekly schedules, likely due to their higher cumulative dose intensity and pharmacodynamic effects. Further support comes from RCTs. A phase III trial by Kellokumpu-Lehtinen et al. [9] comparing biweekly to triweekly docetaxel in CRPC found a longer median time to treatment failure in the biweekly arm (5.6 months vs. 4.9 months, *p* = 0.014), suggesting that altering the schedule can influence treatment durability. Additionally, Petrylak et al. [27], in their landmark trial, reported a median time to progression of 10.1 months with triweekly docetaxel plus prednisone, reinforcing the efficacy of this regimen in delaying progression.

The observed discordance between the higher PSA response rate with weekly docetaxel and the longer time to treatment failure with triweekly dosing has important clinical implications. PSA response reflects early biochemical tumor activity and treatment sensitivity [28], whereas time to treatment failure is a composite outcome influenced by durability of disease control, cumulative dose intensity, and tolerance to ongoing therapy [29]. Weekly docetaxel, administered at lower per-dose intensity, may achieve more frequent cytotoxic exposure leading to early PSA declines, but this effect may be less durable due to reduced cumulative drug exposure over time.

In contrast, triweekly docetaxel delivers a higher dose intensity per cycle, which may result in deeper tumor cytoreduction and more sustained disease control, despite a lower initial PSA response rate [30]. This suggests that PSA response alone may not fully capture long-term therapeutic benefit and reinforces the limitation of PSA kinetics as a surrogate endpoint for treatment durability. Clinically, these findings imply that weekly docetaxel may be preferable for patients prioritizing tolerability or early biochemical response, such as frail or elderly individuals, whereas triweekly dosing may be better suited for patients with adequate performance status in whom prolonged disease control is the primary objective. This trade-off highlights the need for individualized treatment selection based on both short-term response and long-term disease management goals.

Moreover, we also revealed that both biweekly and triweekly docetaxel regimens are associated with a significantly higher incidence of hepatotoxicity compared to the weekly regimen. Specifically, biweekly docetaxel showed an RR of 3.71 (95% CI: 1.41–9.73), while triweekly docetaxel had an RR of 3.21 (95% CI: 1.50–6.89), with low heterogeneity (*I^2^* = 0%). These findings align with previous studies indicating increased hepatotoxicity with less frequent, higher-dose docetaxel schedules. Regarding gastrointestinal toxicity, our analysis demonstrated that triweekly docetaxel is associated with a significantly higher incidence of vomiting compared to the weekly regimen (RR: 2.47, 95% CI: 1.31–4.63), also with low heterogeneity (*I^2^* = 0%). This observation is consistent with findings from a meta-analysis by van Eijk et al. [31], which reported increased gastrointestinal adverse events, including vomiting, in patients treated with triweekly docetaxel compared to weekly regimens. The higher peak plasma concentrations achieved with triweekly dosing may contribute to the increased gastrointestinal toxicity observed. These findings suggest that weekly docetaxel regimens may offer a more favorable toxicity profile concerning hepatotoxicity and vomiting. However, regimen selection should remain individualized based on patient comorbidities, tolerability, and treatment objectives. Our NMA found that triweekly docetaxel was associated with a slightly higher incidence of anorexia (RR 1.04, 95% CI 0.47–2.30) and diarrhea (RR 1.27, 95% CI 0.27–6.01) compared to biweekly and weekly regimens, although these differences did not reach statistical significance and were accompanied by low heterogeneity for anorexia and high heterogeneity for diarrhea. When compared with van Eijk et al.’s [31] findings on Grade 3/4 toxicities, there is notable inconsistency. van Eijk et al. [31] reported a non-significant reduction in severe diarrhea with weekly docetaxel compared to triweekly (RR 0.50, 95% CI 0.22–1.14, *p* = 0.10), and no significant difference in anorexia was documented as a separate outcome, suggesting that anorexia might not be a frequent Grade 3/4 event or was variably reported.

We also observed that triweekly docetaxel was associated with a higher incidence of fatigue, febrile neutropenia, and nausea compared to weekly docetaxel regimens, although the differences were statistically insignificant for some outcomes. Notably, the risk of febrile neutropenia was markedly elevated with triweekly dosing (RR: 12.3), indicating a clinically relevant safety concern despite low heterogeneity among studies. These findings align closely with the systematic review and meta-analysis by van Eijk et al. [31], who evaluated the impact of docetaxel scheduling on treatment tolerability and efficacy in metastatic breast cancer patients. van Eijk and colleagues [31] reported that weekly docetaxel was better tolerated, with significantly fewer hematologic toxicities, including febrile neutropenia, and reduced incidence of fatigue and gastrointestinal side effects compared to triweekly administration. This improved safety profile of weekly dosing was also associated with fewer treatment discontinuations due to adverse events, emphasizing the importance of scheduling in maintaining patient adherence and quality of life during therapy [28].

Clinicians should weigh the trade-offs between efficacy and tolerability when selecting a docetaxel regimen, tailoring the dosing schedule based on individual patient factors such as age, comorbidities, performance status, and treatment objectives. Routine monitoring for adverse effects and proactive management of toxicity are essential to maximize therapeutic benefit while minimizing harm.

### Strength and limitations

This NMA has several notable strengths, including adherence to PRISMA and Cochrane guidelines, prospective registration with PROSPERO, and a comprehensive literature search across major databases without language or date restrictions. The inclusion of both RCTs and high-quality retrospective studies enhances the generalizability of findings, while the use of NMA allows for robust indirect and direct comparisons across weekly, biweekly, and triweekly docetaxel regimens. A wide range of efficacy and safety outcomes were assessed, and study quality was rigorously evaluated using established tools. However, this study also has limitations.

Many studies did not report hazard ratios or provide survival data in non-comparable formats, and substantial variability in outcome definitions and follow-up duration precluded reliable pooling or network comparison.

A key limitation of this analysis is the substantial heterogeneity observed in several pooled outcomes, most notably time to treatment failure (*I^2^* = 96.8%) and anemia (*I^2^* = 91.1%). Such high heterogeneity reduces the precision and reliability of the pooled effect estimates and indicates that the observed summary measures should be interpreted with caution. The direction of effect for these outcomes was generally consistent across studies; however, the magnitude of benefit varied considerably, limiting confidence in the exact size of the estimated effects.

This heterogeneity likely reflects clinical and methodological diversity across included studies, including differences in disease stage (mHSPC vs. mCRPC), docetaxel dose intensity, prior systemic therapies, follow-up duration, and variability in outcome definitions and toxicity grading. In addition, the inclusion of both randomized and observational studies may have further contributed to between-study variability. As a result, while the findings provide useful comparative insights into dosing schedules, conclusions, particularly for outcomes with very high heterogeneity, should be viewed as hypothesis-generating rather than definitive.

### Conclusion

This NMA provides a comprehensive evaluation of the efficacy and safety of weekly, biweekly, and triweekly docetaxel regimens in patients with prostate cancer. Weekly docetaxel demonstrated the highest PSA response rate and a more favorable safety profile, particularly with lower incidences of hepatotoxicity and gastrointestinal side effects. In contrast, triweekly dosing was associated with a significantly longer time to treatment failure, indicating potentially greater disease control, but at the cost of increased adverse events such as febrile neutropenia and fatigue. While triweekly dosing offers longer disease control, clinicians must carefully monitor and manage increased toxicities to maintain the patient’s quality of life. These findings highlight the importance of balancing efficacy with tolerability and underscore the need for personalized treatment decisions based on patient characteristics, comorbidities, and treatment goals. Further high-quality, head-to-head randomized trials are needed to confirm these findings and clarify the optimal dosing schedule in clinical practice. Additionally, incorporating quality of life assessments and cost-effectiveness analyses in future research will be essential to support informed clinical decision-making and guide healthcare policy.

## Abbreviations

ADT: androgen deprivation therapy
CIs: confidence intervals
mCRPC: metastatic castration-resistant prostate cancer
mHSPC: metastatic hormone-sensitive prostate cancer
NMA: network meta-analysis
NOS: Newcastle-Ottawa Scale
PRISMA: Preferred Reporting Items for Systematic Reviews and Meta-Analyses
PROSPERO: International Prospective Register of Systematic Reviews
PSA: prostate-specific antigen
P-scores: probability-scores
RCTs: randomized controlled trials
RRs: risk ratios

## Supplementary materials

The supplementary material for this article is available at: https://www.explorationpub.com/uploads/Article/file/1002360_sup_1.pdf.

## Declarations

### Availability of data and materials

The database for this systematic review was sourced online from databases listed in the methods. Additional data are available from the corresponding author upon request to any qualified researcher.

### Copyright

© The Author(s) 2026.
