# Supplementary material for: Comparison of weekly docetaxel regimens in prostate cancer: a systematic review and frequentist network meta-analysis
Source: Explor Target Antitumor Ther. 2026 Feb 27;7:1002360. doi: 10.37349/etat.2026.1002360 (PMC12950338; doi:10.37349/etat.2026.1002360)
Supplement: Supplementary file 1 [file 1002360_sup_1.pdf]

Supplementary Table 1: Search strategy across included databases

Database	Search String	Results
MEDLINE	(<br>"Prostatic Neoplasms"[MeSH]<br>OR "Prostate Cancer"[tiab]<br>OR "Prostatic Cancer"[tiab]<br>OR "Prostate Neoplasm*"[tiab]<br>OR "Metastatic Prostate Cancer"[tiab]<br>OR "Castration-Resistant Prostate Cancer"[tiab]<br>OR "Hormone-Refractory Prostate Cancer"[tiab]<br>OR mCRPC[tiab]<br>OR mHSPC[tiab]<br>)<br>AND<br>(<br>"Docetaxel"[MeSH]<br>OR docetaxel[tiab]<br>OR taxotere[tiab]<br>)<br>AND<br>(<br>weekly[tiab]<br>OR biweekly[tiab]<br>OR "bi-weekly"[tiab]<br>OR "two-weekly"[tiab]<br>OR fortnightly[tiab]<br>OR triweekly[tiab]<br>OR "three-weekly"[tiab]<br>OR schedule*[tiab]<br>OR dosing[tiab]<br>OR regimen*[tiab]<br>OR administration[tiab]<br>OR dose-intensity[tiab]<br>)	1,176
Embase	('prostate cancer'/exp OR 'prostate carcinoma':ti,ab OR 'metastatic prostate cancer':ti,ab OR 'castration resistant prostate cancer':ti,ab) AND ('docetaxel'/exp OR docetaxel:ti,ab OR taxotere:ti,ab) AND (weekly:ti,ab OR biweekly:ti,ab OR 'two weekly':ti,ab OR fortnightly:ti,ab OR triweekly:ti,ab OR 'three weekly':ti,ab OR dosing:ti,ab OR regimen*:ti,ab OR schedule*:ti,ab)	2,538
Cochrane CENTRAL	(prostate cancer OR prostatic neoplasm) AND (docetaxel) AND (weekly OR biweekly OR two weekly OR fortnightly OR triweekly OR three weekly)	176

Supplementary Table 2: Evaluation of the quality of the 7 included cohort studies by using the Newcastle-Ottawa Scale

Studies	Selection				Comparability		Exposure or outcome			No. of star
	1	2	3	4	1	2	1	2	3	
Berthold et al. (2008)	*	*	*	*	*		*	*		7
Kim et al. (2017)	*	*	*	*	*		*	*		7
Martinez-Recio et al. (2022)	*	*	*	*	*	*	*	*	*	9
Samar et al. (2020)	*	*	*	*	*		*	*		7
Sayin et al. (2022)		*	*	*	*		*	*		6
Shimura et al. (2020)	*	*	*	*	*		*	*		7
Yuk et al. (2024)	*	*	*	*	*	*	*	*	*	9

Supplementary Table 3. Identifying local inconsistency via indirect versus direct comparisons and the use of a node splitting approach.

Outcome	Comparison	*p*-value
PSA Response Rate	2:1	0.7874
	3:1	0.5070
	2:3	0.1670
Time for treatment failure or progression	2:1	**< 0.0001**
	3:1	**< 0.0001**
	2:3	NA
Hepatotoxicity	2:1	0.4600
	3:1	NA
	2:3	0.4600
Vomiting	2:1	NA
	3:1	NA
	2:3	NA

Any Adverse Events	2:1	0.8830
	3:1	0.8830
	2:3	NA
Thrombocytopenia	2:1	NA
	3:1	NA
	2:3	NA
Anemia	2:1	NA
	3:1	NA
	2:3	NA
Arthralgia	2:1	NA
	3:1	NA
	2:3	NA
Neuropathy	2:1	0.3301
	3:1	0.5244
	2:3	0.2037
Neutropenia	2:1	NA
	3:1	NA
	2:3	NA
Fatigue	2:1	0.8587
	3:1	0.5412
	2:3	0.3718
Febrile neutropenia	2:1	NA
	3:1	NA
	2:3	NA

Nausea	2:1	NA
	3:1	NA
	2:3	NA
Diarrhea	2:1	NA
	3:1	NA
	2:3	NA
Anorexia	3:2	NA

Note: Weekly, biweekly, and triweekly docetaxel therapy are referred to as 1, 2, and 3, respectively. P-values lower than 0.05 indicate that local inconsistency was observed in the specific comparison. Statistically significant p-values presented in bold. NA represents that there is either no direct or indirect evidence available for that specific comparison.

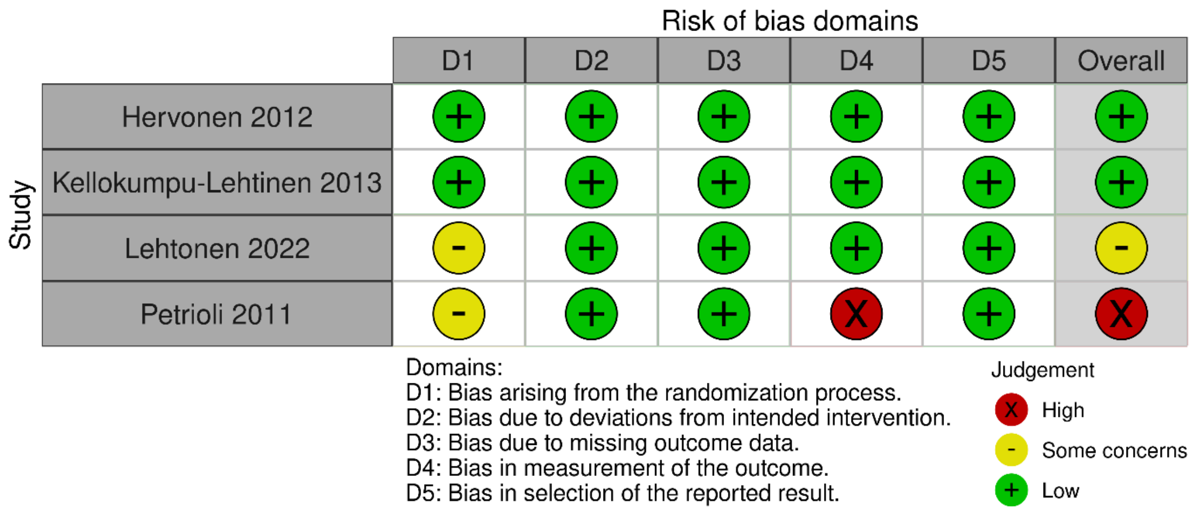

Supplementary Figure S1: Traffic plot of Risk of Bias Assessment using ROB 2.0

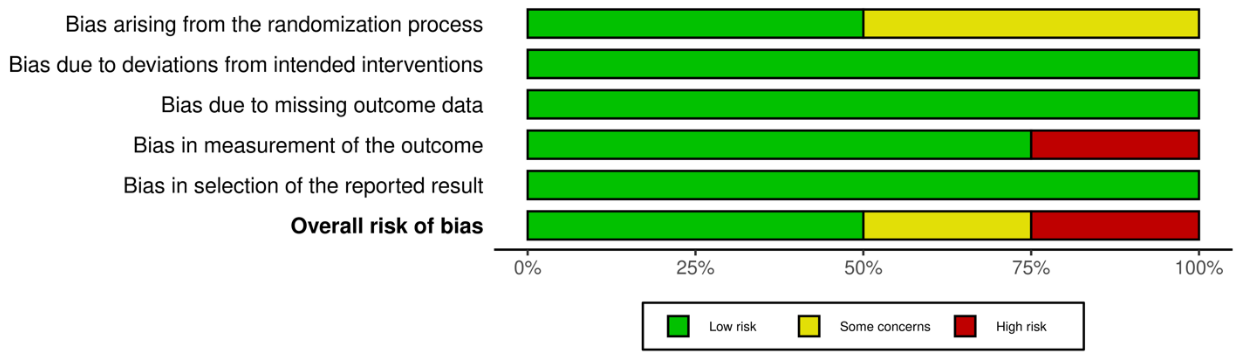

Supplementary Figure S2: Summary Plot of Risk of Bias Assessment

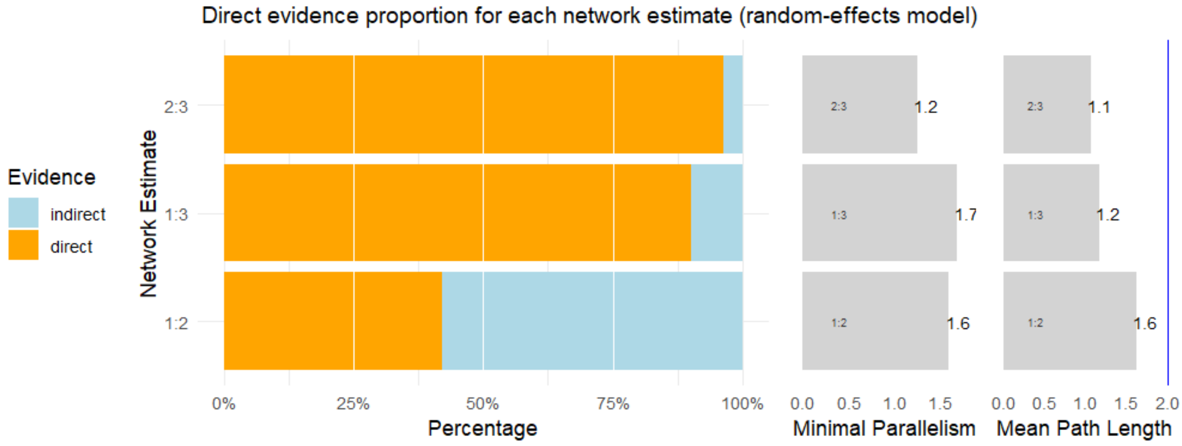

Supplementary Figure S3: Netweight plot for PSA

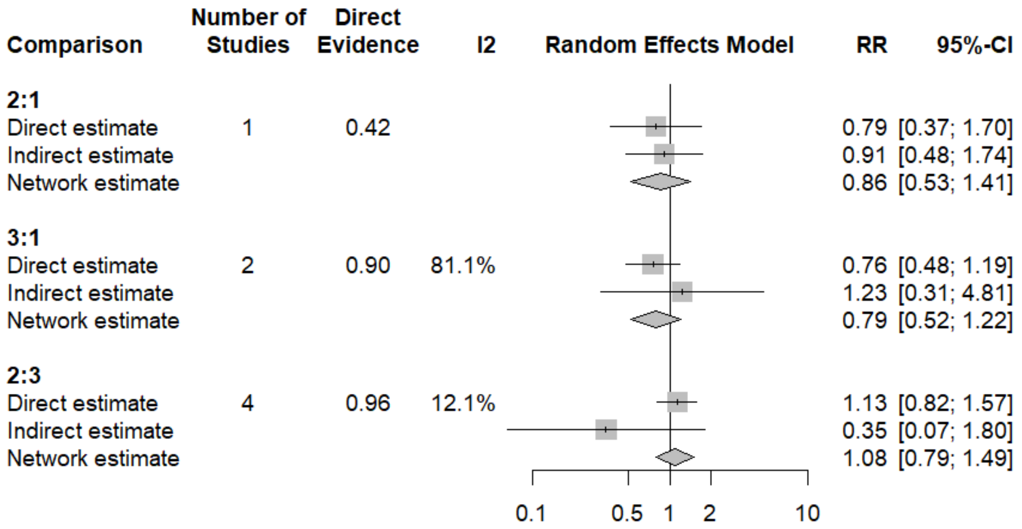

Supplementary Figure S4: Netsplit plot for PSA

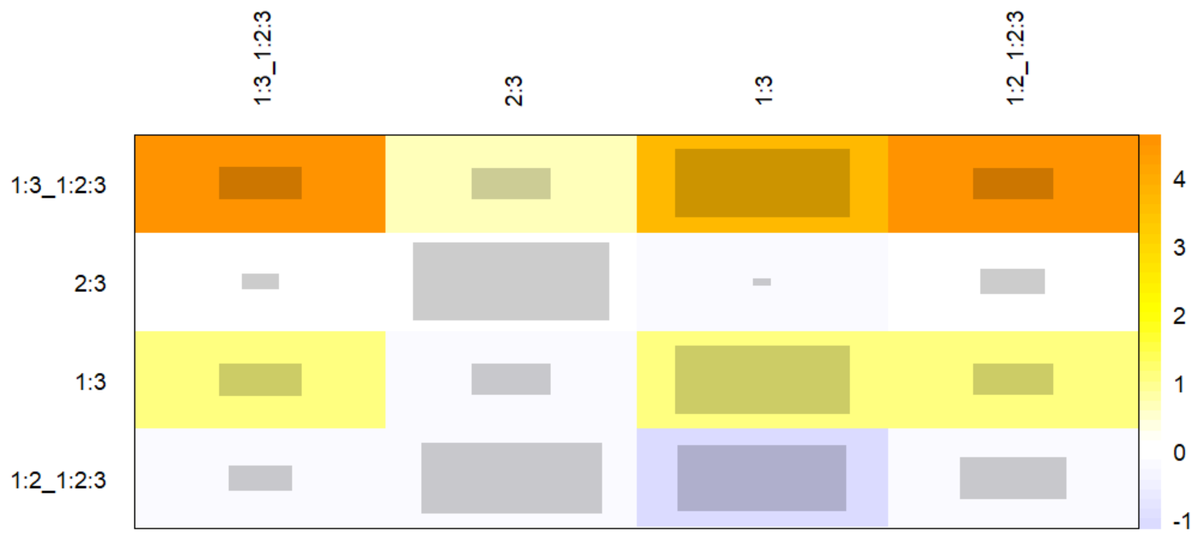

Supplementary Figure S5: Netheat plot for PSA

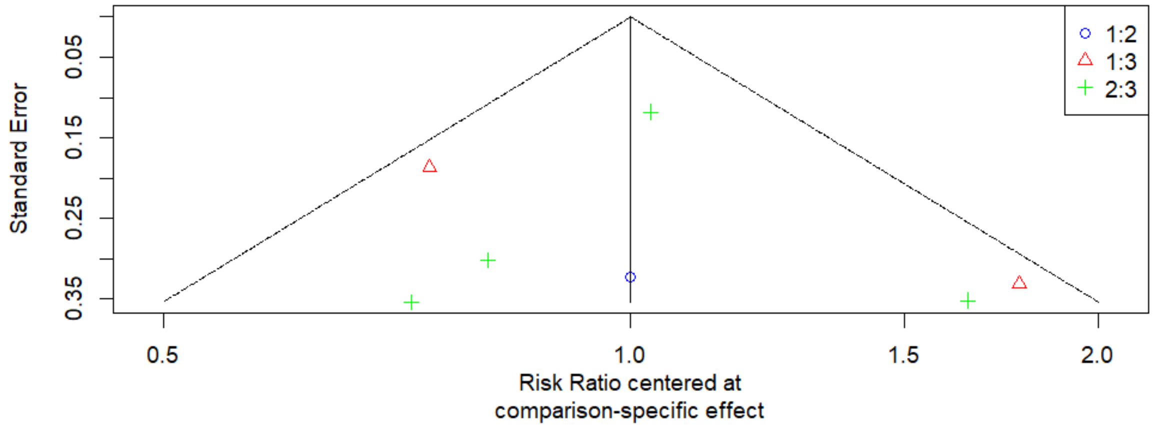

Supplementary Figure S6: Funnel plot for PSA

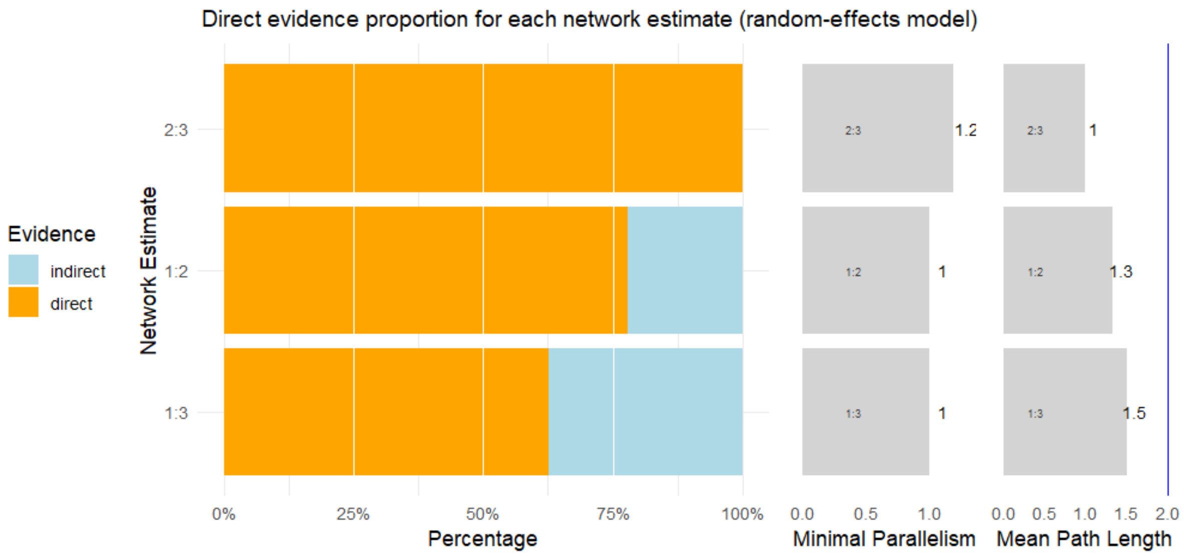

Supplementary Figure S7: Netweight plot for time to treatment failure/progression

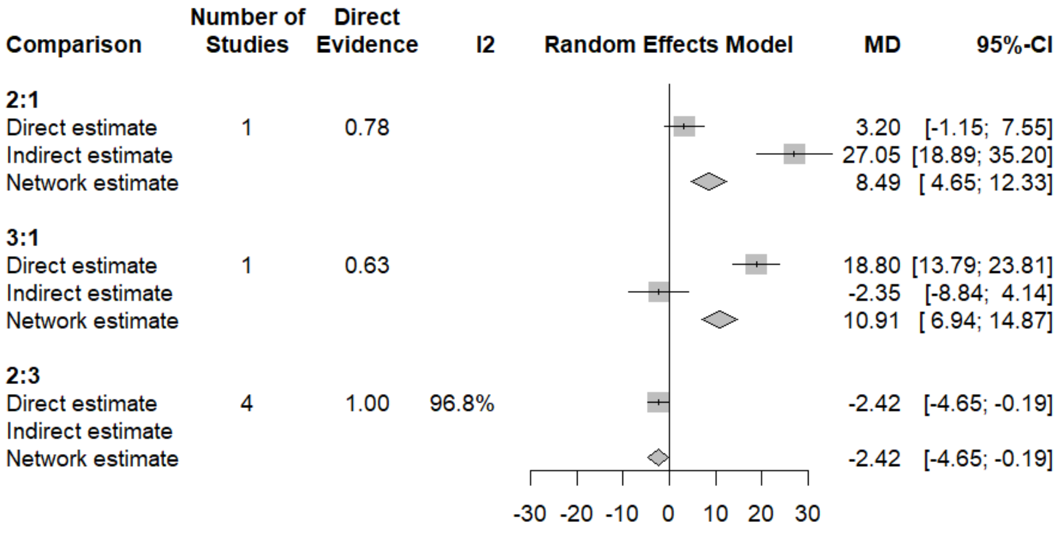

Supplementary Figure S8: Netsplit plot for time to treatment failure/progression

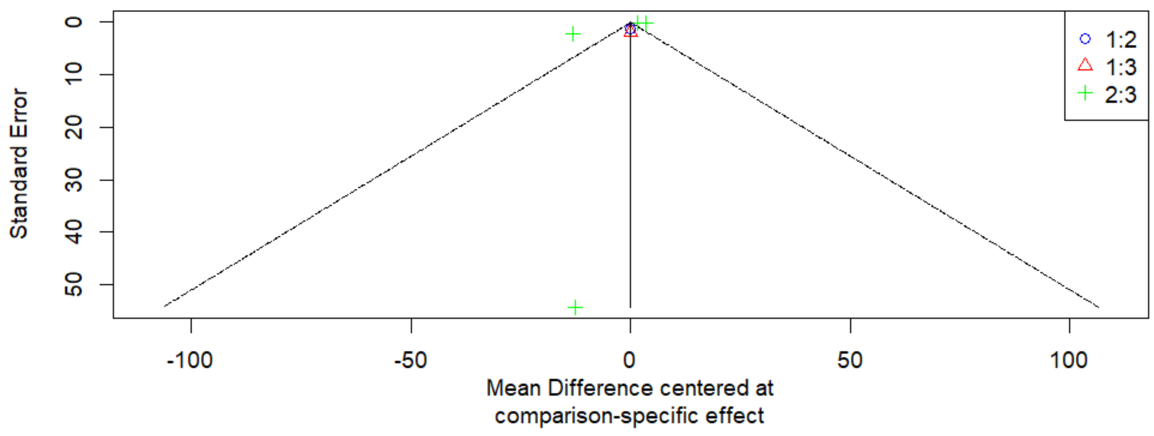

Supplementary Figure S9: Funnel plot for time to treatment failure/progression
